# Factors Determining Consumer Acceptance of Biofortified Food: Case of Zinc-Fortified Wheat in Pakistan's Punjab Province

**DOI:** 10.3389/fnut.2021.647823

**Published:** 2021-06-09

**Authors:** Muhammad Rizwan, Yueji Zhu, Ping Qing, Debin Zhang, Umar I. Ahmed, Hui Xu, Muhammad A. Iqbal, Abdul Saboor, Arshad M. Malik, Adnan Nazir, Xuelian Wu, Puming He, Azam Tariq

**Affiliations:** ^1^School of Economics and Management, Yangtze University, Jingzhou, China; ^2^Changjiang River Belt Economic and Development Research Institute, Yangtze University, Jingzhou, China; ^3^Management School of Hainan University, Haikou, China; ^4^College of Economics and Management, Huazhong Agricultural University, Wuhan, China; ^5^College of Public Administration, Huazhong Agricultural University, Wuhan, China; ^6^Department of Agribusiness and Applied Economics, Muhammad Nawaz Shareef University of Agriculture Multan, Multan, Pakistan; ^7^Institute of Agricultural and Resource Economics, University of Agriculture Faisalabad, Faisalabad, Pakistan; ^8^Department of Economics and Agri. Economics, PMAS Arid Agriculture University, Rawalpindi, Pakistan; ^9^Department of Agricultural Economics, Sindh Agriculture University Tando Jam, Tando Jam, Pakistan; ^10^College of Humanities and Social Sciences, Huazhong Agricultural University, Wuhan, China

**Keywords:** biofortification, zinc-fortified wheat, sensory factors, determinants to accept, probit model, Pakistan

## Abstract

Zinc (Zn) is a fundamental micronutrient required by all living organisms. Zn deficiency among children under 5 years, pregnant, and child-bearing women has been identified in developing countries such as Pakistan. Biofortified crops can increase micronutrient levels and decrease deficiencies. Meanwhile, consumer acceptance is essential, given that genetic alterations can occur during biofortification, resulting in changes in sensory traits and the quality of grains. Therefore, the present study focuses on the determining factors for consumer acceptance of Zn-biofortified wheat., an experimental survey was conducted to achieve the study's objectives. Qualitative and quantitative data were collected and analyzed from 203 respondents in the Punjab province. The results regarding sensory perceptions revealed that people attached great importance to the appearance of the chapati prepared with Zn-biofortified wheat. Therefore, they were willing to purchase Zn-biofortified wheat when asked to choose between the conventional wheat and the Zn-biofortified wheat. Moreover, the probit model illustrates that the level of education in the family and having young children aged under 5 years in the household positively impacted the acceptance of Zn-biofortified wheat among the participants. The findings suggest that there is significant scope for promoting Zn-biofortified wheat in the country. It is also imperative to ensure its availability across various regions so that households with weak purchasing power can buy and address their Zn deficiency. Furthermore, policymakers could introduce reforms targeting business communities for food management, keeping Zn-biofortified wheat in the priority stream.

## Introduction

According to the global nutrition report, the global burden of malnutrition worsened in 2020, with one in nine individuals experiencing starvation and one-third being considered obese (1). Many households are expected to be further affected by 2050 (2). Due to micronutrient deficiencies, diet-related problems can lead to chronic diseases, affect the quality of life, and increase healthcare costs (3). Various organizations are focusing on the United Nations (UN) Sustainable Development Goals (SDGs) aimed at ending all forms of hunger and promoting good health and well-being (4, 5). For instance, non-governmental organizations (NGOs), namely, Acumen, Save the Children, Plan International, Brac, and Care, are working on SDG-1, i.e., no poverty. Heifer International, KickStart, and One Acre Fund are focusing on SDG-2, i.e., zero hunger. Similarly, International Committee of the Red Cross, Helen Keller International, Partners in Health are working for SDG-3, i.e., good health and well-being. Hence, many other NGOs are supporting various SDGs (6). The lack of access to a nutritious diet for pregnant women and children below the age of 2 could result in health issues affecting mental or physical growth (7). Micronutrient deficiency is one of the main factors affecting human health on a large scale (8). Numerous strategies have been implemented to reduce malnutrition, including diversification of food, food supplementation, and biofortification in crops, especially staple food crops. It has analyzed that biofortified foods can minimize nutrition deficiency among men and women of all ages. It also significantly impacts reducing disease burden, particularly Disability Adjustment Life Years (9, 10).

Zinc is recognized as one of the common deficiencies among deprived societies in emerging economies (11). Furthermore, it is revealed that the death of children under the age of 5 is associated with Zn deficiency globally (12). According to a previous study, it found that 901.4 million population in Asia, 236.8 million in Africa, and 135.6 million in Latin America and the Caribbean people are under Zn deficiency risk (13). At the same time, 45% of children aged between 1 and 9 years old are under Zn risk in Africa (14). Moreover, Zn deficiency causes stunting among children (15) and deprived cognitive growth, which is alarming (16, 17).

It is estimated that more than 100 million people in Pakistan suffer from malnutrition (18). Moreover, 40% of children aged below five are stunted in growth, 18% sustained with wasting, 29% are underweight, and 10% are obese (19). Zn deficiency is prevalent in 18.6% of children aged below five and in 22% of women during reproductive age (between 15 and 49 years old) in the country. The deficiency is higher among rural children and women compared with urban areas (19). Efforts to address Zn deficiency were made through different approaches in developed and developing countries, including Pakistan. For instance, supplements of micronutrients were used to enhance Zn levels under various conditions (20). However, this practice was expensive and unsuitable for pregnant women. Therefore, the food fortification approach has been broadly adopted. Furthermore, previous studies found that Zn biofortification in wheat has improved grain quality and caused to reduce the Zn deficiency among people (21–28). However, past literature has shown that economic efficiency is lower due to Zn deficiency, thereby increasing the mortality rate. Another study conducted in India (27) found that high-zinc wheat flour (HZWF) positively impacted illness and pneumonia by about 17%, and vomiting was controlled in 40% of children. It also positively influenced fever among 9% of pregnant and child-bearing women.

Further, food helps in improving health and well-being. In response, food producers have been offering new products that can match the socioeconomic status of individuals (29). Consumer acceptance and farmer adoption of biofortified staple crops determine technology coverage. The breeding process through conventional methods or genetic alteration is called biofortification, for instance, golden rice and Zn-biofortified wheat. Staple crops should focus on biofortification through conventional breeding methods, given that consumers have strictly rejected genetic alteration (30). Considering that biofortified staple crops are different in color, taste, and texture compared with traditional staple crops, there is a need to raise awareness among farmers to ensure acceptance. Maize enhanced with beta carotene is a typical example of biofortified staple food. An earlier study by Stevens and Nelson analyzed consumer acceptance of maize fortified with pro-vitamin in Mozambique (31). The researchers used taste checks and trading experiments and found that participants preferred traditional maize over biofortified orange maize, owing to its appearance. The study also found that although the participants liked and expressed their willingness to consume meals prepared with orange biofortified maize, they continued to prefer traditional maize. They also stated that participants in urban areas such as Maputo agreed to accept biofortified orange maize, probably because of increased knowledge and awareness regarding the importance of micronutrients.

Another study conducted in Kenya found that participants preferred traditional white maize compared with yellow biofortified maize in the western regions. At the same time, those in the Siaya district liked biofortified yellow maize than traditional white maize (32). Furthermore, another biofortified staple crop, cassava, has been fortified with beta carotene. A study conducted in Brazil noted that participants welcomed biofortified cassava during a survey conducted using a hypothetical crop (30). Meanwhile, sweet potato has been widely used as a staple food in developing and developed countries. It has revealed that respondents selected orange fortified sweet potato during choice experiments in Uganda (33).

Furthermore, an earlier study highlighting consumer acceptance of Zn-biofortified rice in Latin America found that biofortified rice had higher acceptability than traditional rice in terms of appearance and taste, and grain quality characteristics (34). In comparison, a recent study conducted in Australia explored the acceptance of white and red wheat. The study revealed that participants preferred red wheat over white wheat variety in terms of taste. However, white wheat has a relatively higher amount of micronutrients like Zn, iron, phosphorus, and potassium (35). Similarly, another study revealed that Zn-biofortified wheat has higher Zn nutrients in flour (36). Thus, Zn-biofortified wheat varieties having a high-yielding potential can bring in more economic and health benefits to farmers and society overall (37). However, there might be low acceptability among consumers due to alteration in color, flavor, and other features in the new variety. Wessells and his colleagues conducted a study on the Zn fortification of cereal flours and recommended that researchers analyze the level of Zn fortification demand (38). Poole and his colleagues stated a need to be more focused on agriculture-nutrition research to introduce the best biofortified staple food crops to cope with micronutrient deficiency (39).

Zn-biofortified wheat was introduced in Pakistan, with official trials commencing in 2016. However, wheat or wheat flour with added Zn was not available in the open market until the survey of the present study (i.e., 2018). Although a few studies have highlighted the importance of biofortification, there is limited information on consumer behavior and the factors affecting the acceptance of biofortified wheat in Pakistan. According to existing literature and the best of our knowledge, only one study was conducted in Khyber Pakhtunkhwa (KPK) to examine public insight before commencing a randomized controlled trial to explore the efficacy of consuming Zn-biofortified wheat flour for curtailing Zn deficiency (40). However, their methodology and approach were different, and they inquired and collected data on community/Jirga-based. Therefore, it is imperative to conduct an in-depth study to gauge people's willingness to accept Zn-biofortified wheat individually in the country. Hence, the present study is designed to address the question as to what are the sensory and socioeconomic factors that affect consumers' acceptance of Zn-biofortified wheat.

## Methodology

### Study Sites

**Figure 1:**
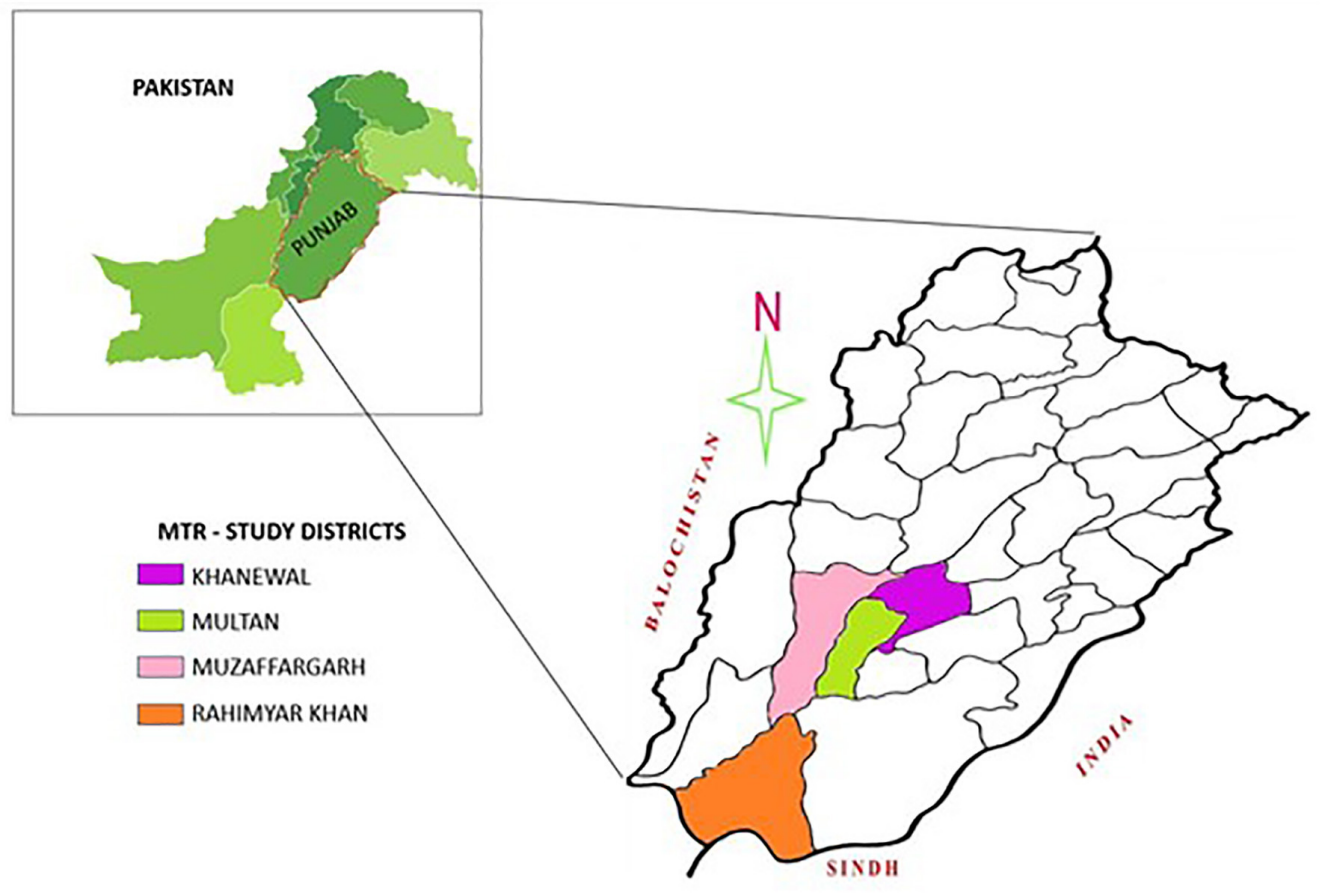
Map of the study area.

The present study was conducted in 2018 in Punjab Province, the largest province by population and the second-largest by area. Four districts, namely Khanewal, Multan, Muzaffargarh, and Rahimyar Khan, were selected from the province's southern region (see Figure 1). These districts were selected given that southern Punjab is less developed and less educated than upper Punjab (41). Most of the population lives below the poverty line and has poor economic and health conditions. Potable water is not accessible to many people in the area, while women and children are malnourished due to a lack of nutritious food (19).

### Socioeconomic Factors

The socioeconomic status of the participants was investigated and recorded. These attributes include age, education, total members in the household, and numbers of children under 5 years. In addition, respondents' weekly consumption of selected food items (i.e., meat, eggs, fish, chapati prepared with wheat flour, and vegetables) was also recorded. According to the respondents' consumption patterns, dietary diversity (DD) was categorized into three variables and analyzed. The first variable included all types of meat, including fish and eggs, the second variable included all types of vegetables, while the third variable included wheat flour. The respondents were asked whether they used these food items during the previous week. Due to financial and time constraints, the DD factors were analyzed using dummy variables, where 1 denotes they consumed DD products during the last week, and 0 denotes if they have not. DD is the technique used to address the micronutrient deficiencies and enhance the nutrients among the population (42, 43).

### Design Experiment of the Study

The present study used an experimental survey methodology to analyze the factors influencing consumer acceptance of Zn-biofortified wheat, and 203 respondents participated were in the experiment trial. Participants were informed about the purpose before the start of the experiment. The participants were aware that the results of the experiment would be used only for educational research purposes. Verbal consent was also taken from them. Moreover, they were assured that their information and data would be kept secure and safe and used only for educational purposes. The experimental design and whole procedure were presented in the committee meeting where it was confirmed and approved by committee members.

For analyzing the contingent value, the respondents were offered an opportunity to engage in real trading rather than a hypothetical survey instrument. Our study design follows Diamond and Hausman (44), which avoids bias for data collection. To attract participants, a field experiment was designed in the market where customers are available without bias. This experiment provides a real understanding of the consumers owing to their market experience such as trade, including buying and selling activities to decide on real settings (45, 46). However, in this experiment, the bidding procedure for the market setting was limited due to budget constraints. In our study, we used the perceptions technique to gauge sensory factors. Furthermore, the respondents were independent to conduct trade or exchange conventional wheat flour with the Zn-biofortified wheat flour, as per the design experiment.

Since this research is designed to enhance the number of respondents, a certain amount of biofortified Zn flour was made accessible from a farmer in Rahimyar Khan for the experiment with the help of local farmers due to not availability of Zn-biofortified wheat or flour in the market. Accordingly, two types of wheat flour bags (2 kg each with both types of wheat flour varieties) and pamphlets were distributed. One bag had Zn-biofortified wheat flour, and the other had conventional wheat flour. The flour bags were priced at Rs. 100 PKR (equal to US$1), and the flour price was determined according to the fine flour in the market price at the time of data collection.

While entering the marketplace, a research team member from the data collection team provided detailed information to the respondent by elucidating the purpose of the study, describing the nutritional benefits of Zn-biofortified wheat, and presenting the processes to follow it. Another fellow of the research team demonstrated the experiment, and the trial continued through voluntary participation. Moreover, procedures were followed to previous studies conducted by Meiligard et al. and Tomlins et al. (47, 48). Each person was given a chapati to taste or at least one-fourth of a chapati of each variety. Both types of chapatis were cooked traditionally. Therefore, we included only those participants who voluntarily agreed to taste the chapatis cooked using both types of wheat flour varieties (conventional and Zn varieties) and excluded those who tasted only the chapatis prepared with conventional wheat flour.

After consuming both varieties of chapatis, they were asked to give a score for each type by using a 5-point Likert scale: 1 for “very bad” and 5 for “very good” in terms of taste and overall acceptance. Additionally, for texture, appearance, and aroma, the respondents were asked to rank the chapatis using similar Likert scoring. Information was also provided to the respondents that Zn-biofortified wheat was more nutritious than the conventional variety to enhance awareness of the nutritional aspect. However, the participants were concerned that zinc biofortified wheat is new and may have health risks. Therefore, after the data collection team member gave them information about Zn values, only the people who voluntarily agreed to participate in this experiment were added. The questionnaire proforma was also translated into the national language to understand the questions and answer them easily.

At the end of the experiment regarding sensory perception, a 2-kg bag of Zn-biofortified wheat flour was provided to each of them. Subsequently, the data collection team members asked them to either keep this flour or to trade it with local wheat flour, and the data about their trading behavior were recorded.

## Results and Discussion

### Descriptive Statistics and Sensory Perceptions

The results regarding participants' demographic and sensory perceptions are shown in Table 1. Results reveal that males accounted for 57% of overall participants. The age of the participants ranged from 15 to 68 years old, with an average value of 36. The educational level of the respondents varied from illiterate to 16 years of schooling, having a mean value of 7.2 years. It indicates that the average education of participants is below the middle school level. The results also showed that the number of members in participants' households ranged from two to nine persons, with six being the average value. These results show that most households comprise six family members. On average, the participants had one child under the age of five, and the maximum number of these children was three.

**Table 1:** Descriptive statistical analysis.

**Variables**	**Description of variables**	**Mean**	**Std. dev**.	**Minimum**	**Maximum**
Gender	If male Male=1, and female=0	0.0.57	0.05	0	1
Age	Age of respondent in years	36.42	14.20	15	68
Education	Schooling years	7.28	0.05	0	16
Family size	Numbers of members in family	6.21	0.06	2	9
Children <5 years	Numbers of Children under 5 years in a family	1.31	0.02	0	3
Meat/eggs/fish	If consumed last week=1 otherwise=0	0.0.67	0.43	0	1
Vegetable	If consumed last week=1 otherwise=0	0.0.83	0.19	0	1
Wheat flour	If consumed last week=1 otherwise=0	0.0.99	0.01	0	1
Wheat conv. appearance	Likert scale, 1=very bad, 5=very good	4.31	0.97	1	5
Wheat conv. taste	Likert scale, 1=very bad, 5=very good	4.17	1.11	1	5
Wheat conv. texture	Likert scale, 1=very bad, 5=very good	4.19	1.09	1	5
Wheat conv. Aroma	Likert scale, 1=very bad, 5=very good	3.63	1.34	1	5
Wheat Zn appearance	Likert scale, 1=very bad, 5=very good	4.56	0.84	1	5
Wheat Zn taste	Likert scale, 1=very bad, 5=very good	4.01	1.15	1	5
Wheat Zn texture	Likert scale, 1=very bad, 5=very good	4.21	1.11	1	5
Wheat Zn aroma	Likert scale, 1=very bad, 5=very good	3.89	1.23	1	5
Trade conv. vs. Zn wheat	If want to trade conv. with Zn wheat=1 otherwise=0 with information about Zn wheat	0.0.62	0.33	0	1

Additionally, the results revealed that 67% of the respondents had consumed meat, fish, and eggs during the last week of data collection^1^, while 43% of households' members did not use meat food items. Similarly, 83% of consumers had consumed vegetables in the previous month. The data also showed that 99% of respondents consumed wheat flour during the last month, given that wheat is a staple food in the study area. In an earlier study, a 1% increase in DD was associated with a proportional increase in income (49). Therefore, DD data were collected and considered to understand the nutritional levels of the participants.

The results regarding the sensory characteristics of wheat varieties revealed that there was not much difference in mean scores for conventional and Zn-biofortified wheat. The results regarding appearance show that mean scores 4.31 and 4.56 were given to conventional and Zn-biofortified wheat, respectively. It indicates that Zn-biofortified wheat is given a marginally high preference compared with conventional wheat. In contrast, a little more priority is given to conventional wheat as compared with Zn-biofortified wheat concerning its taste, as shown in Table 1. It could be attributed to respondents' taste buds getting habituated to the conventional variety. Similarly, texture and aroma mean scores for conventional wheat were 4.19 and 3.63, respectively. While for Zn-biofortified wheat, these scores were 4.21 and 3.89, respectively. Many of the respondents (62%) were willing to consider trading conventional wheat with Zn-biofortified wheat.

### Distribution of Sensory Perception Rating

The results regarding sensory perceptions rating distribution are presented in Table 2. The results reveal that people gave higher scores to the appearance of the chapati prepared with Zn-biofortified wheat and a higher taste score to conventional wheat variety. Most respondents preferred conventional wheat for texture, while the majority liked Zn-biofortified wheat chapati concerning aroma. Overall, the results show that the respondents are willing to accept the Zn-biofortified wheat variety, although consumers' perceptions toward this wheat varied for different sensory factors. Similar results were found in two other studies, one conducted on rice in Latin America and another conducted in KPK, Pakistan (34, 40). For instance, Woods et al. revealed that participants showed a willingness to accept the biofortified Zn rice 035 variety than the local rice variety. Likewise, Mahboob et al. reported in their study results that participants showed motivation to accept the biofortified wheat flour. In addition, we applied paired *t*-test to analyze the mean difference for a better understanding of sensory perceptions and values, as shown in Table 2. Results show that participants are willing to accept Zn-biofortified wheat flour regarding appearance and aroma sensory factors. While taste and texture have no significant values for Zn-biofortified wheat flour.

**Table 2:** Distribution of sensory perception ratings based on Likert scale.

**Sensory factors**	**Percentage rating**						***t*-test**
	**Very bad**	**Bad**	**Okay**	**Good**	**Very good**		
Appearance	Conventional	1.5	3.5	19.3	25.4	50.3	−0.345^***^
	Zn biofortified	1.6	3.9	14.6	27.1	52.8	
Taste	Conventional	0.0.8	1.8	19.5	25.4	52.5	0.059
	Zn biofortified	3.5	8.6	23.4	22.5	42.0	
Texture	Conventional	1.8	5.3	16.8	26.9	49.2	0.034
	Zn biofortified	2.9	6.8	25.6	24.9	39.8	
Aroma	Conventional	2.6	13.5	25.9	29.8	28.2	−0.241^*^
	Zn biofortified	8.4	11.6	21.2	24.3	34.5	

*** *Indicate significance at <1%, and*

* *indicate significance at 10%*.

### Results of Multivariate Analysis

The sensory perception data presented in the previous section and Table 2 suggest that many participants in the study area, particularly urban areas, are open to accepting Zn-biofortified wheat. The results of previous studies support these findings (50). Therefore, multivariate model analysis was applied to understand the factors that influence acceptance comprehensively. A probit model is used to achieve this objective and followed the previous methodology applied by Chib and Greenberg; and Czado (51, 52). The generalized probit model is as follows.

$$\begin{array}{l} \text{Pr}\left(Y=1/X=x\right)=\;\Phi x^{\prime }\beta \end{array}$$

The dependent variable used in this model is the trade of conventional vs. Zn-biofortified wheat. If the participants agree to trade, it has a value of 1; otherwise, it is 0. The independent variables used in this model are gender, age, education, family size, children under the age of five, DD, and sensory variables.

The results of the probit model are demonstrated in Table 3. The results illustrate that education positively impacts all three types of quadratic, i.e., full, reduced quadratic, and without DD. Furthermore, it shows that educated participants are more likely to accept Zn-biofortified wheat than less-educated respondents. Previous studies showed similar results and supported our study findings (14, 53–56). For instance, Tumuhimbise et al. in 2013 and Siwela and his colleagues in 2020 conducted studies. They illustrated in their study that education through the community about nutrition positively influences consumers' decision to accept biofortified food. Further, Kuchenbecker and his colleagues stated in 2017 that nutrition could be enhanced among children aged between 6 and 23 months by providing nutrition-based education through community channels (54).

**Table 3:** Probit model results.

**Variables**	**Full quadratic**	**Reduced quadratic**	**Without dietary diversity**
Gender	0.291	0.269	0.341
	(0.323)	(0.398)	(0.321)
Age	0.053	0.008	0.103
	(0.246)	(0.562)	(0.681)
Education	1.007^**^	1.901^*^	0.095^*^
	(0.002)	(0.029)	(0.092)
Family size	−0.263	−0.179	−0.145
	(0.059)	(0.269)	(0.293)
Children <5 years	0.0685^***^	0.296^**^	0.321^**^
	(0.019)	(0.008)	(0.007)
Meat/fish/eggs	4.494	0.514^*^	0.356
	(0.106)	(0.081)	(0.22)
Vegetable	0.416^*^	0.305^*^	0.371^*^
	(0.076)	(0.072)	(0.028)
Wheat flour	0.129	0.021	0.028
	(0.320)	(0.637)	(0.552)
Wheat Zn appearance	1.342^**^	1.248^**^	1.179^*^
	(0.005)	(0.009)	(0.012)
Wheat Zn taste	0.363	0.179	0.145
	(0.159)	(0.269)	(0.293)
Wheat Zn texture	0.007	0.008	0.008
	(0.823)	(0.386)	(0.412)
Wheat Zn aroma	3.967^*^	3.830^*^	3.901^*^
	(0.035)	(0.038)	(0.036)
Constant	2.617	1.938^*^	0.904^*^
	(0.059)	(0.039)	(0.098)
*R*^2^	0.569	0.438	0.385

* *p < 0.1*,

** *p < 0.05, and*

*** *p < 0.01*.

Moreover, the results revealed that a big family size had a negative coefficient value in all three cases but not significant. It indicates that having more family members may lead to budget constraints, due to which such families are unwilling to accept Zn-biofortified wheat. However, households having children aged below five are ready to accept innovative food items such as Zn-biofortified wheat. It implies that parents of children under 5 years of age are more inclined to accept Zn-biofortified wheat for their children's health. Previous studies conducted in different countries are in line with these results and support our study (33, 57). The variable fish, egg, and meat consumption is positively significant, and the respondents are motivated toward accepting Zn-biofortified wheat. It shows that DD has an impact on the acceptance of new food. In addition, respondents who consumed vegetables have also shown an interest in accepting the Zn-biofortified wheat variety.

In the present study, considering the sensory impact on the acceptance of Zn-biofortified wheat, the results illustrate that the appearance and aroma of this wheat variety have a positive influence. It indicates that consumers prefer Zn-biofortified wheat due to its appearance and aroma. Although the coefficient value of taste for Zn-biofortified wheat has positive, it does not appear significant. It may be because consumers do not appreciate the taste of novel food as much as the conventional variety they are accustomed to and have been using for a long time. Previous studies elucidated that sensory factors are crucial for accepting novel food (33, 57–59). For instance, Birol and his colleagues conducted a study in 2015 in African, Asian, and Latin American countries to examine consumer acceptance of biofortified food. They illustrated that sensory factors are crucial for encouraging the consumption of biofortified crops among consumers.

## Conclusions and Policy Recommendations

The current study reveals consumers' attitudes toward Zn-biofortified wheat and the determining factors for its acceptance. Accordingly, 203 participants were enrolled to participate in the experimental research activity to evaluate the acceptance of Zn-biofortified wheat from Punjab province in Pakistan. Considering that Zn-biofortified wheat has been newly launched in Pakistan to enhance Zn levels in the diet of the country's population, there is limited data for analysis. Therefore, the data was generated by the experimental processes. The results regarding sensory perceptions rating distribution illustrate that participants liked Zn-biofortified wheat in the context of appearance and aroma. In contrast, conventional wheat scored more concerning texture and taste. While taste factor found non-significant to Zn-biofortified when they asked for trading with conventional wheat. Moreover, when the participants were asked to trade choices, i.e., conventional to Zn-biofortified wheat, many were ready to get Zn-biofortified wheat. In addition, socioeconomic factors were also found to influence Zn-biofortified wheat acceptance among participants. For instance, education, having children aged below 5, and the monthly consumption of vegetables were positively significant. The findings recommend that Zn-biofortified wheat has a scope of acceptance in Pakistan. Therefore, efforts should educate the people about the significance of this variety, and its availability must be ensured in the market. The present study may help policymakers and other stakeholders design and define strategies and promote better health policies.

## Limitations of the Study

Our study has few limitations, which are as follows: (i) Due to financial and time constraints and unavailability of data, this study generated the data through an experiment and covered the southern area of Punjab province, a less-developed area of the province, and analyzed 203 participants. Hence, to understand the population's perceptions in greater detail, the next research should conduct, and the data would be collected from more respondents in the country. (ii) Moreover, this study only focuses on the acceptance of Zn wheat. Maybe the people were inclined to accept Zn-biofortified wheat because it was the same price as the conventional wheat. Hence, further research can be conducted on willingness to pay for Zn-biofortified wheat.

## Data Availability Statement

The raw data supporting the conclusions of this article will be made available by the authors, without undue reservation.

## Ethics Statement

The studies involving human participants were reviewed and approved by College of Economics and Management, Huazhong Agricultural University China. For the present study, all participants were informed about the purpose prior to starting the experiment that the results of the experiment will be used only for educational research purpose and verbal consent was taken from the respondents due to avoid the participants' hesitation though they were free to take part or not in the experiment. Moreover, they were assured that their information and data will be kept secure and safe and used only for education purposes. Only volunteers were selected for experiment of the present study and kept their recorded data.

## Author Contributions

MR and UA: data curation and software. MR, AN, and XW: formal analysis. MR, AN, and MI: investigation. MR, DZ, PH, HX, YZ, and DZ: resources. MR, UA, XW, and AT: data entry. MR and MI: validation. MR, PH, HX, and YZ: visualization. MR, AS, AM, MI, and UA: writing—review and editing. MR, PQ, and HX: supervision. All authors have read and approved the final version of the manuscript.

## Conflict of Interest

The authors declare that the research was conducted in the absence of any commercial or financial relationships that could be construed as a potential conflict of interest.

## Footnotes

^1^It is worth noting that these data were collected in winter, and are likely to vary during other months.
